# Security-by-Experiment: Lessons from Responsible Deployment in Cyberspace

**DOI:** 10.1007/s11948-015-9648-y

**Published:** 2015-04-21

**Authors:** Wolter Pieters, Dina Hadžiosmanović, Francien Dechesne

**Affiliations:** Delft University of Technology, CyberSecurity@TUDelft, P.O. Box 5015, 2600 GA Delft, The Netherlands; University of Twente, Services, Cybersecurity and Safety, P.O. Box 217, 7500 AE Enschede, The Netherlands; 3TU.Ethics @ Eindhoven, Eindhoven University of Technology, P.O. Box 513, 5600 MB Eindhoven, The Netherlands

**Keywords:** Adversarial experiments, Cyber security, Empirical security, Responsible experimentation, Security-by-experiment, Social experiments

## Abstract

Conceiving new technologies as social experiments is a means to discuss responsible deployment of technologies that may have unknown and potentially harmful side-effects. Thus far, the uncertain outcomes addressed in the paradigm of new technologies as social experiments have been mostly safety-related, meaning that potential harm is caused by the design plus accidental events in the environment. In some domains, such as cyberspace, adversarial agents (attackers) may be at least as important when it comes to undesirable effects of deployed technologies. In such cases, conditions for responsible experimentation may need to be implemented differently, as attackers behave strategically rather than probabilistically. In this contribution, we outline how adversarial aspects are already taken into account in technology deployment in the field of cyber security, and what the paradigm of new technologies as social experiments can learn from this. In particular, we show the importance of adversarial roles in social experiments with new technologies.

## Introduction

### Safety and Security

The paradigm of new technologies as social experiments (henceforth NTaSE) deals with responsible deployment of technologies that may have undesirable side-effects. The idea is that it is impossible to identify all potential problems in the design stage, and that it may therefore be necessary to subject society to a deployment experiment with uncertain outcomes.

Thus far, the uncertain outcomes addressed in the NTaSE-paradigm have been mostly safety-related (nuclear waste and accidents, health effects of nanoparticles, genetically modified crops etc.). Safety implies that potential harm is caused by the design plus accidental events in the environment, such as natural disasters and human mistakes. What the paradigm has *not* covered so far are so-called adversarial risks. These are risks that are not caused by probabilistic natural events or accidents, even human failures, but rather by the determined, strategic behaviour of an adversarial agent. This is often labelled security as opposed to safety.

The most obvious example in the NTaSE literature of a technology with a security component is nuclear technology: adversaries who get hold of nuclear material may use it for weapons. Recently, Lehtveer and Hedenus (2015) discussed this extensively in terms of nuclear proliferation. Where the NTaSE-literature has discussed nuclear technology, it has focused on accidents (safety), and has not addressed this adversarial perspective (security). For example, Krohn and Weingart (1987) explicitly focus on the “accident as implicit experiment”, analysing the Chernobyl Meltdown, and in the same vein, Van de Poel (2011) analyses the Fukushima accident. Finally, Taebi et al. (2012) address four characteristics of nuclear energy that complicate traditional risk assessment (low probabilities with large consequences, uncertainty and ignorance, long term effects, intense emotions by both proponents and opponents), but do not include the potential effects of intentional adverse behaviour.

In general, the possible use of technologies for military purposes is termed dual use. However, security can be broader than that when considering use by *enemies* or *adversaries*, with opposing and conflicting goals, who need not always be of military type. New technologies may be used for weapons, but also for fraud, other criminal activities, or morally problematic behaviour that is not in itself illegal (yet). The NTaSE literature has not yet addressed security aspects for other new technologies with potential dual use-issues and other relevant adversarial risks either. The work on biotechnology and nanotechnology so far mainly focuses on responsibility: Robaey (2013) address distribution of responsibility for GM-risks through the philosophical notion of ownership; Jacobs et al. (2010) propose conditions for the responsible marketing of nano(bio)tech products. Thus, we conclude that the security perspective is currently underrepresented within the paradigm.

### Cyber Security

Adversarial risks are also key in the domain of cyber security. Cyber security is a relatively new field of research and technology, which aims at protecting information technology and connected infrastructures against malicious actions by adversaries (hackers, terrorists, nation states engaged in cyber warfare). In this domain, many technologies have been deployed that allowed adversaries to take advantage. In some cases, benevolent people or groups have pointed developers at weaknesses, which have or have not been fixed subsequently.

We believe that the deployment of many new technologies may induce adversarial risks, next to accidental risks, that are not to be neglected. Dealing with these adversarial components has not yet been highlighted as a systematic part of the NTaSE framework, and it requires an extension of the framework to accommodate the adversarial perspective. In that respect, we think that the domain of cyber security contains important lessons for the NTaSE paradigm. In order to demonstrate this, we provide an overview of selected cyber security problems and solutions from the NTaSE perspective. In particular, we discuss the issue of *adversarial roles* in different interpretations of *cyber security as social experiment*.

This paper is a further elaboration and adaptation of ideas initiated in Pieters et al. (2014a). In that paper, we highlighted the relevance of the NTaSE paradigm *for* the cyber security community, by discussing how the conditions for responsible experimentation can be interpreted for experiments in the context of cyber security. In contrast to the previous paper, the present paper aims at identifying lessons that NTaSE can learn *from* cyber security. Therefore, the focus in this paper is on developments in cyber security that can be seen as social experiments, what techniques have been used to improve deployment processes in the light of adversarial risk, and also what hasn’t worked, in terms of problematic cases. The sections on experimental practices (“Adversarial Experiments in Cyber Security” section) and deployment techniques (“Techniques for Responsible Deployment” section) are entirely new.

### Structure of this Paper

In section “Deployment Problems”, we will discuss several occasions in which the deployment of security-sensitive information technologies had the characteristics of a social experiment, but generally was not designed as such. These examples point to several possible “adversarial roles” in such histories. In section “Adversarial Experiments in Cyber Security”, we discuss how adversarial roles have been embedded in more formal experimental practices related to cyber security, such as software testing but also manipulative experiments. In section “Techniques for Responsible Deployment”, we discuss techniques in the cyber security domain that have been used to implement responsible deployment, in particular related to monitoring and feedback, from which we may be able to draw lessons for other technology domains in which adversarial risk plays a role. In section “Conditions for Responsible Deployment” we discuss relevant conditions for responsible deployment in the light of adversarial risks, and we conclude in section “Conclusions and Discussion”.

## Deployment Problems

To illustrate the issue of responsible deployment in relation to cyber security, we discuss three examples. These examples point to undesirable effects that the deployment of new technologies in the cyber domain may cause, and how potential problems have been identified in the course of the deployment experiments. The examples are electronic voting, smart electricity meters, and a public transport chipcard, all in the Dutch context.^1^

### Electronic Voting

One example of the deployment of a security-sensitive new information technology is the introduction of electronic voting in the Netherlands (Jacobs and Pieters 2009; Pieters 2008; Pieters and Van Haren 2007). In the Netherlands, electronic voting machines had been introduced since the early 1990s. After the introduction of the machines, regulations were not revisited or updated, nor was there any renewed evaluation of the risks.

When—as one of the last municipalities—Amsterdam introduced electronic voting in 2006, a pressure group was founded to fight electronic voting. Their main argument in favour of a return to paper voting was the ability of citizens to observe and verify the procedure. In electronic voting, one cannot see or deduce that “what goes in is what comes out”, unless one trusts the workings of the machines, which is precisely what is at stake here. The pressure group had an excellent media strategy, and achieved coverage of their manipulation of machines they had bought. Basically, they showed that it would be easy for anyone having access to the machines to replace the chips containing the counting programs with fraudulent ones. Such access is available both to manufacturer personnel as well as to those involved in transport and storage. Besides, storage security was shown to be low, giving external attackers the opportunity to gain access as well. Also, and more or less by accident, they demonstrated that it was possible to eavesdrop on the choice of the voter by means of a radio antenna, capturing signals emitted by the device while showing the voter’s choice [TEMPEST attack, Gonggrijp and Hengeveld (2007)].

Because of the information-leak-by-radiation, the certification of the touch-screen machines was suspended before the 2006 elections. Commissions were installed to study both the past and the future of electronic voting. The “past” commission concluded that the government had too easily outsourced their responsibility for the electoral process, and requested that the government take control again (Hermans and Twist 2007). The “future” commission proposed a combination of ballot printer and vote counter, where the intermediate votes would be readably printed on paper (Election Process Advisory Commission 2007). However, because an expert group concluded that the radio signal problems could not be solved for any device on which the voter chooses a candidate, also the ballot printer was deemed infeasible. It was therefore decided to abandon all forms of electronic voting. Currently, future electronic voting possibilities are discussed, again based on the framework of ballot printer plus vote counter, based on a report by another commission (Commissie onderzoek elektronisch stemmen in het stemlokaal 2013).

### Smart Meters

The EU Directive on energy efficiency (2006/32/EG) prescribed the installation of smart energy meters that provide end users with information on their actual use, so that they can contribute to energy savings. In the Netherlands, a combination of two separate legal bills was proposed in 2008, amounting to the mandatory roll-out of smart meters, which were still to be developed. These meters were to send measurements for gas (hourly), and electricity (quarter-hourly) to the *network operators*, who would forward this information to the *energy providers*, who could then inform the *consumers* about their consumption. The initial proposal also included signalling functions (to detect energy quality), switching functions (to remotely switch off in case of non-payment or disasters) and regulatory functions (to add options to the meter) for the *network operators*. In fact, some energy providers had already started to provide households with smart meters upon request (e.g. Oxxio from 2005).

After the assessment by the Dutch privacy watchdog, the proposal was amended by requiring explicit consumer consent for transferring detailed consumption data to energy suppliers (however, daily usage would be mandatorily forwarded). Also, addition of purpose specification and use limitation, data subjects’ right of access, data removal after use, and suitable security measures were required according to the Dutch privacy law (Cuijpers and Koops 2013). The October 2008 report by the Consumer Union concluded that smart meters also put pressure on the right to inviolability of the home, and the right to respect for family life (Hoenkamp et al. 2011).

On the basis of this analysis, the Dutch Senate rejected the bills in 2009, and adopted an adapted version, that allowed for conducting pilot projects (*Proeftuinen*) involving smart meters. Users, providers and operators experimented with smart grids and meters on a voluntary basis, in selected neighbourhoods. The aim was trying out incentivising users to conserve energy, and to participate in balancing the grid under the presence of sustainable energy sources whose production depends on weather conditions (Cuijpers and Koops 2013). In the meantime, to resolve the issues raised by the Senate, a broad stakeholder collaboration came to define the so-called *Dutch Smart Meter Requirements* (DSMR4), that implement the adapted version of the bill (in particular, it specifies the last point: defining data granularity for each task). The abolition of the detailed readings was considered to take “the largest privacy sting out of the Dutch law” (Cuijpers and Koops 2013).

Interestingly, the pilot projects have, as far as we have found (Dechesne 2013), not been exploited to explicitly experiment with the effectiveness of the new requirements with respect to the security and privacy issues that were raised when the law was rejected. The pilots are mostly focused on testing the functionality of the technology, and learning how to deal with human participation in balancing the grid. Questions about privacy and security, and associated requirements and values, were not asked to the consumers. In October 2014, a new proposal for the broad smart meter roll out in the Netherlands, on a voluntary basis, was approved by parliament.

User participation in the electricity net is a great paradigm shift, both for users and operators. Experience shows that wrong assumptions are easily made about tasks, responsibilities and risks with respect to (cyber) security. For example, operators are used to thinking in top-down controllable components, which made them neglect privacy issues for consumers, while users are not used to be conscious about the electricity flow, let alone to adapt their behaviour – they need incentives. The pilots that are conducted provide a good opportunity for both sides to learn in a relatively controlled environment how roles in the system may shift, and what that would mean for the risks and responsibilities with respect to cyber security. The lack of explicit attention to (cyber) security and privacy in the smart grid pilots (Dechesne 2013) leaves room for reflection on how the pilots could have been used to learn about these aspects for smart metering, by consciously designing them as social experiments.

### OV-Chipkaart

The “OV-chipkaart” is a public transport chipcard gradually rolled out in the Netherlands between 2002 and 2012. The initial version of the card used the MIFARE classic chip. The cryptographic algorithm used to protect the card contents was kept secret by the producer. In 2007, German researchers revealed part of the secret algorithm by so-called reverse-engineering (Nohl et al. 2008).

In 2008, Dutch researchers of Radboud University Nijmegen found two possible attacks on the card. The attacks would enable them to read, clone, or restore cards by retrieving the cryptographic keys. The researchers demonstrated this possibility with several cards used in practice, including the OV-chipkaart. However, travelling with cloned cards would still be detected by the back-end system in place.

The researchers informed the government and the manufacturer, with publication of the results anticipated 7 months later. The idea of this “responsible disclosure” was giving the responsible authorities enough time to address the issue before the knowledge would become public. The manufacturer then asked the court to prohibit publication.^2^ The university claimed their actions were reasonable from the point of view of academic freedom, and the court ruled in the university’s favour. The results were published eventually (Garcia et al. 2008).

After these events, the organisation responsible for the OV-chipkaart set up a scientific advisory board to enable better handling of feedback in the future. The MIFARE classic card was also gradually replaced with a different one, using standard rather than proprietary cryptography.

### Analysis

In the above examples, it is clear that the problematic aspects of these “social experiments” do not lie in threats to health, environment or safety. Although harm may ensue in domains where information systems are connected to critical (physical) infrastructures, the primary concern is the regulation of access, in this case to information and information systems. In an electronic voting system, there has to be an acceptable balance between who can influence the results, who can verify the results, and who can learn what somebody voted. In the smart metering case, there is a similar question on what data is collected, and who can use what data for which purpose. In the OV-chipkaart case, the possibility of (monetary) fraud by means of manipulation of data was the key issue. Privacy and integrity, next to availability, are core concerns.

Several authors have discussed this fundamental premise of information and cyber security (regulating access) in different terms: ontological friction (Floridi 2005), causal insulation and perimeters (Pieters 2011), and order machines (Vuorinen and Tetri 2012). Security controls are aimed at making it easy for some to get access while at the same time making it difficult for others. This is meant to ensure that the opportunities for adversaries/attackers/criminals gaining illegitimate benefit out of the deployed technology are limited. This is also discussed in the field of crime science, where regulating access is operationalised in terms of making crimes less attractive from an adversary point of view: increasing effort, increasing risk (for the adversaries), reducing rewards, reducing provocations, and removing excuses (Dechesne et al. 2014; Gradon 2013).

For each attempt to regulate access, the question can be raised how effective it is (Pieters et al. 2014b), which will in turn provide some information on expected harm, as well as the potential of unfair gain for the adversaries. Typically, mathematical reasoning, tests, and experiments can help answer this question. Mathematical reasoning can prove properties of cryptographic tools, testing can reveal security flaws in software, and social experiments (in the narrow social science sense) can provide information on human defences, such as compliance with security rules and guidelines. In many cases, the question on the adequacy of the combined digital, physical and social access controls is still a matter of expert judgement, although risk management tools may help.

In the cases, we have seen several instances in which new information on the security quality of the systems was revealed by external parties after deployment, based on their own assessments. These can be seen as instances of “security-by-experiment”, in contrast to the “security-by-design” approaches often advocated. This raises the question which techniques are already available for experimenting with security both in the design and in the deployment stage.

In the next section, we will look into experimental practices on the effectiveness of regulating access *before* deployment. We will then use this as a basis for discussing practices on evaluating and improving regulation of access *after* deployment, as in the NTaSE paradigm.

## Adversarial Experiments in Cyber Security

Where the traditional paradigm of new technologies as social experiments focused on safety, the application to security-sensitive technologies requires taking into account other aspects of the notion of “experiment”. In this case, the uncertain outcomes are not only due to natural events and human mistakes, but also to strategic behaviour of adversaries, or attackers.

Experimental approaches differ for adversarial and non-adversarial contexts. This does not only hold for new technologies as social experiments, but also for experiments in a narrower sense, i.e. observing the responses of systems to controlled conditions. To understand the consequences for the notion of new technologies as social experiments, in this case cyber security as social experiment, it is therefore helpful to investigate existing experimental approaches, in the narrower sense, in cyber security. Typically, those are controlled studies on the effect of adversarial behaviour on systems, which can involve technical as well as human components.

### Security Testing

One such context occurs in security testing, which is quite different from regular testing of information technology. For functional testing, i.e. testing whether the behaviour of the system conforms to the specification, it is tested whether the right behaviour occurs under the specified conditions. If I want to send a secret from A to B, I can observe whether the secret submitted at A is actually produced at B. What I cannot observe is who else might have learnt the secret in the meantime.

It is widely accepted in the security community that security is not a functional property. Although certain aspects of security can be embedded in functions, such as authentication, in general security requirements say something about what should *not* happen in a system, and not in particular on the relation between inputs and outputs. An electronic voting system may produce the right result for any sequence of input votes submitted through the intended channel (and therefore be functionally correct), but that does not mean that it is secure (as votes may also be input in non-standard ways).

This also means that security flaws do not show up in regular software testing procedures, unless specific tests for security are executed. For example, a database may return correct results for all “normal” queries, but a specifically crafted “odd” query may allow an attacker to retrieve sensitive information he is not authorised for, or even to delete (parts of) the database (so-called SQL injection). Lack of input validation is one of the important causes of security weaknesses (Tsipenyuk et al. 2005), and it requires tests that intentionally provide “odd” inputs.

Even when specific security testing is in place, the sheer number of possible security flaws may cause the testers to overlook important aspects. For example, side-channel attacks make use of measurements on power consumption, timing, or electromagnetic radiation to deduce properties of the information being processed in a system (Standaert et al. 2009). This may for example allow an attacker to eavesdrop on voter’s choices in an election using electronic voting machines, as we have seen in the electronic voting case (Pieters 2009). Security testing thus requires *taking the perspective of the adversary* to see what tests should be executed (Rennoch et al. 2014). This requires some idea about possible adversary behaviour.

Although components may be tested separately, taking the adversarial perspective happens in particular on a system level, where so-called “penetration tests” can be executed to find paths through which attackers can gain access to critical system assets. In penetration tests, one hires ethical hackers to try and break into the system, reporting the results to the system owner. These hackers play the role of the adversaries in the tests, relieving the system owners from having to play this role themselves.

### Security Awareness Experiments

In the above, we have explained how adversarial testing is different from functional testing, focused on information systems in the technical sense, and illustrating the adversarial aspects. However, humans often play a very important role in the protection of information and connected infrastructures, and this aspect can be subjected to tests or experiments as well. Adversarial roles are therefore also important in experiments on so-called social engineering.

Social engineering (Tetri and Vuorinen 2013) refers to the exploitation of human weaknesses or lack of awareness to gain access. Phishing attacks, in which fake e-mails request user credentials, are the most widely known example. Social engineering may also involve face-to-face interaction or phone calls, in which the victim is asked for specific information that can be used by the attacker. To this end, the attacker may pretend to be someone else, such as a system administrator (impersonation). Social engineering is thus fundamentally tied to the manipulation of people.

Because of this, associated experiments inevitably have elements of manipulation and persuasion, which makes them different from standard experiments. Social science studies on manipulation have a long history, and go back to the notorious Milgram experiments, in which obedience to authority was measured. In the experiment, participants were asked to administer high voltage shocks to a fictive person (Milgram 1974). In security, such “adversarial experiments” are essential to test for the feasibility of executing attacks by manipulating people. Experiments have been conducted with phishing e-mails (Finn and Jakobsson 2007), and handing keys over to strangers (Bullée et al. 2015). Another variant is the digital equivalent of lost letter experiments, measuring the pick-up rate of dropped USB keys (Lastdrager et al. 2013), which could contain malware. Penetration testing has also been generalised to include social engineering, allowing interaction between the penetration testers and employees of the organisation being tested (Dimkov et al. 2010).

In adversarial experiments, adversarial roles need to be assigned. This can be penetration testers, people designing phishing e-mails for the experiment, or people asking a favour from others in social engineering setups. In controlled experiments, the adversarial roles are carefully scripted, to make sure the experiment is realistic, respectful, reliable, repeatable, and reportable (Dimkov et al. 2010). In penetration tests, the penetration testers typically have more freedom to try out scenarios that they think might work. Although such tests may show that weaknesses exist in complex systems, they are less repeatable (etc.) than controlled experiments. Other participants are typically not aware of the adversarial roles, to make sure they behave as they would in a natural setting.

### Analysis

In various different contexts, social as well as technical, experiments are different when they try to take security into account. This is because one needs to consider the adversaries in the experiments. Contrary to a safety context, a single weakness may be sufficient for low security, because adversaries behave strategically, and direct their efforts towards weak spots in the system. Adversarial behaviour therefore needs to be “designed” into the experiments.

When broadening the experiments to socio-technical systems rather than technical systems, another concern is the moral acceptability of the experiments. Adversarial experiments with human subjects require special care, because (i) they can typically not be informed about the true purpose of the experiment and need to be debriefed afterwards, (ii) this may cause additional negative effects within the experiment, and (iii) the subjects may be punished for their behaviour after the end of the experiment, for example by their company, because they “did the wrong thing”. Most people would no longer consider the Milgram experiment acceptable, and the acceptability question should also be asked for adversarial experiments in security.

What does the notion of adversarial roles in experiments imply for the NTaSE paradigm? Most of the experimental techniques (in a narrow sense) outlined above provide general information about the susceptibility of people to social cyber attacks (social engineering experiments), or about a specific technology when it is being developed (security testing). After deployment, the environment is often even less controlled. Still, under the assumptions of the NTaSE paradigm, there will be side-effects that only show up after deployment. In a security context, adversarial roles will be needed to find such effects. We will first investigate existing approaches for responsible deployment in cyber security, and then discuss implications for the NTaSE paradigm.

How does the cyber security community leverage the notion of adversarial roles in security experiments in the deployment context?

## Techniques for Responsible Deployment

In this section, we take social experiments in cyber security out of the lab and into the real world. After deployment of security-sensitive technologies in cyberspace, what methods are in place to gather security-relevant information, in particular related to security weaknesses, and who play the adversarial roles in such experiments? And which conditions for responsible experimentation (Van de Poel 2009) do these techniques relate to?

### Beta Versions

Many software vendors distribute so-called “beta versions” prior to official release. These versions allow interested users/early adopters to get an early experience with the new features, while at the same time knowing that they may still contain mistakes. Before the software is rolled out for a wider audience, mistakes that are found can be fixed, limiting the potential harm that such mistakes could cause. This is an instance of the condition of *consciously scaling up*.

For systems involving hardware, such as the cases discussed earlier, it is typically natural to scale up gradually. However, what is problematic is that security often does not play a role in the decision to scale up. For example, we have identified that security was hardly accounted for in Dutch smart grid pilots (Dechesne et al. 2014). If security is not taken into account, security issues may show up later, in particular because larger-scale systems are more interesting for (cyber) attackers.

### Open Source

Another way to organise feedback on software is to publish the source code, i.e. the program in a human-readable programming language from which the machine-executable code is derived (Hoepman and Jacobs 2007; Payne 2002). By allowing others to see the source code, they may be able to spot and report mistakes, also those with potential security consequences. A further benefit is that actors independent of the original developer may continue the development and maintenance of the software if the original developer is unable or unwilling to do so.

Open source enables better *monitoring* and *feedback* with respect to the deployed technology. The effect of open source is typically dependent upon the willingness of external parties to inspect the code and report issues.

Many newer systems for electronic voting are required to be open source by the responsible governments, in order to improve monitoring, feedback, and continuity. Still, open source does not guarantee that this particular software is actually running on the machines, which is a tricky technical question.

### Bug Bounties

Bug tracking systems are widely used for the reporting and fixing of mistakes found in software. These systems may be confidential, but are typically public in case of open source code.

In bug bounty programs, software companies pay those who find mistakes in their software, which often have security consequences (Böhme 2006; Just et al. 2008). The idea is that users, or “white-hat” hackers, have a greater incentive to report the vulnerabilities if there is a reward attached. This becomes increasingly important in the context of black markets, in which the same vulnerabilities could be sold to “real adversaries”, in particular when the vulnerability is unknown and there is no patch [zero-day, Bilge and Dumitras (2012)]. There is typically a relation between the severity of the bug and the amount paid.

More generally, guidelines for what is called “responsible disclosure” have emerged (Cavusoglu et al. 2005), for example when scientists find issues in widely deployed technologies, as in the OV-chipkaart case. It is considered appropriate to give the vendor a reasonable amount of time to resolve the issue before going public.

Bug bounties and responsible disclosure ensure *feedback* in systems with adversarial risk, when systems are too complex to test completely before deployment. The monetary aspect (payment) is particularly important when there are other markets for identified weaknesses. The payment may incentivise actors like the pressure group in the e-voting case and the Nijmegen researchers in the OV-chipkaart case, but in particular less benevolent ones, to (a) perform such studies in the first place, and (b) report the results to the problem owners first. Thereby, they make it more likely that security weaknesses are found and resolved before they can be exploited.

### Red-Team–Blue-Team/Gaming

When the goal is to make system operators more aware of security issues, a red team/blue team training exercise may be considered (Mirkovic et al. 2008). In this model, half of the participants play attacker roles whereas the other half play defender roles, with respect to a simulated system or the real system. The attackers try to find possible attack vectors, whereas the defenders try to block those. Next to training effects, the proceedings of the red team may also point to previously unknown attack strategies, providing *feedback* opportunities. This approach has also been applied outside the computing domain (Grayman et al. 2006). The gamification aspect makes this approach attractive to participants. Still, even a low-profile “argumentation game” already provides opportunities for adversarial roles in risk assessment (Prakken et al. 2013).

This strategy will also increase long-term *monitoring* capability by raising awareness among personnel.

### Honeypots

Honeypots (Kreibich and Crowcroft 2004) are parts of information systems that look like they have important functions, but in fact are only meant to deflect attackers. In this way, they serve two purposes. Firstly, they make it harder for attackers to find the real targets of their attack. Secondly, they gather information about the behaviour of the deflected attackers. This information may in turn be used to optimise the defences of the real assets.

In this way, honeypots are a means to implement the *monitoring* condition in computer systems with adversarial risks, and at the same time to *contain hazards* by increasing efforts for the adversaries. With respect to monitoring, the outcome of evaluations of the experiment is dependent on the behaviour of real adversaries, which may be both an advantage and a disadvantage. On the one hand, one does not need to question whether assigned adversarial roles are representative for the real adversary community. On the other hand, there is no guarantee that any useful information whatsoever will be obtained, as one has no control over the adversaries.

### Socio-Technical Penetration Testing

Penetration tests may focus on technical vulnerabilities, such as software flaws that enable the attacker to have his own code executed, but they may also include physical attack vectors (access to the premises) or social engineering. In this case, it is important to follow ethical guidelines in order to avoid harm (Dimkov et al. 2010).

One particular scientific case study with such approaches involved the retrieval of distributed laptops by students, basically “stealing back” the laptops (Dimkov et al. 2010). In this particular context, it was both meant as an evaluation of security controls and as a training exercise (red team) for the students. In reality, professional penetration testers assist organisations in such tests.

For many systems now deployed, technical penetration tests (white-hat hacking) will be part of the procedure. However, including also the socio-technical context (physical access and social engineering) in the tests is not obvious, as such tests are generally more difficult because of social and ethical concerns.

### Analysis

The abovementioned approaches show the spectrum of responsible deployment techniques in the cyber security domain. One of the main questions in relation to these different approaches is *who plays the adversarial roles* (Table 1). Such roles can be played by real adversaries (honeypots), professional testers (penetration testing), operators (red team/blue team), or users/crowdsourcing (bug bounties). This has consequences for the type of information that is provided about the system, as well as the learning effects.

**Table 1:** Responsible deployment methods per adversarial role type

Adversarial role type	Example method	Example usage
Users	Beta versions	Software
Users	Open source	Software
Users	Bug bounties	Software
Users	Responsible disclosure	Software, systems
Operators	Gaming	Systems, organisations
Professional testers	Penetration testing	Systems, organisations
Real attackers	Honeypots	Computer networks

Based on the above examples, we can conclude that the notion of adversarial roles is an important feature in responsible deployment in the cyber security domain. As we have argued in our earlier paper (Pieters et al. 2014a), the cyber security field does not generally understand these deployment techniques as instances of responsible deployment or NTaSE. For the cyber security community itself, the NTaSE paradigm thus helps to make better decisions on responsible deployment, complementing security-by-design with security-by-experiment.

A specific issue for the cyber domain is the very short update cycle of software. Because of frequent updates, experimental knowledge obtained for one software version may already lose its value when the next version becomes available. These service aspects of software require different perspectives on responsible design than traditional products and architectures (Pieters 2013). Conversely, whereas bug bounties and responsible disclosure work for software, because patches can be distributed quickly, they may be less effective in case of hardware, since it is not possible to change, say, the type of OV-chipkaart on short notice.

Most of the above approaches implement conditions on the design of responsible experiments: monitoring, feedback, conscious scaling up and containment of hazards. It needs to be assumed that preconditions for responsible experimentation have been fulfilled (Van de Poel 2009), specifically absence of alternative testing methods. In particular, offline tests of the systems should have been conducted where possible. While we won’t discuss proportionality and controllability here [see Pieters et al. (2014a) for a short discussion], we do want to point out that informed consent is another important precondition.

NTaSE could learn from cyber security by showing ways to include adversarial roles in responsible deployment. Generalisations of the cyber security practices discussed in this section can provide techniques to include security and adversarial aspects in the paradigm. The obvious question is to what extent these lessons can indeed be applied to other security-sensitive domains, for example nuclear energy. Some approaches will lend themselves better than others. For example, it may be unrealistic to open source plans of nuclear facilities, but gamification of security risk in such facilities would be possible. The differences between physical, digital and social/institutional aspects of technology deployment are key here, and many questions can be asked in terms of whether responsible deployment technique X works for domain Y. Can we make bug bounties work for institutional arrangements rather than software, such as government policies? Or can we create “social honeypots” by fake employee profiles to deflect social engineering attempts?^3^

Even with such techniques, security aspects inevitably change the experimental settings of the deployment, and therefore require reconsidering the conditions for responsible deployment in NTaSE.

## Conditions for Responsible Deployment

In our previous paper (Pieters et al. 2014a), we reinterpreted the conditions for responsible deployment of new technologies can be seen to apply in a cyber security setting. For example, we pointed out that controllability now also applies to adversary behaviour—an additional concern for the condition of controllability—and that scaling-up makes a system more attractive for adversaries—an additional concern for the condition of consciously scaling-up. We refer the interested reader to the abovementioned paper.

Here, we are addressing the generalisation of lessons from responsible deployment in the cyber security domain to other technologies. For general adoption of security considerations in the NTaSE framework, we now discuss the conditions for responsible deployment that we think are most interesting in the light of adversarial risks.

### Informed Consent and Debriefing

Typically, openness on the goals and design of an experiment would be needed to make sure participants understand and agree with the setup. This also holds for social experiments with new technologies. However, knowledge of the design of adversarial aspects may influence the behaviour of the participants, thereby reducing the value of the experiment. For example, if I know that penetration tests will be executed, I will be less likely to comply with the assigned adversaries, because the knowledge has made me more aware.

This issue has already been discussed in the context of penetration testing, and debriefing is seen as an important solution when informed consent is not fully possible. However, that is not the whole story, because it assumes professional adversarial roles. In settings where adversarial roles are played by others than professionals, several other issues emerge. What if real attackers enter the scene? What information should participants in pilots receive in order to prevent harm to, say, their personal data? And if users or operators are allowed to play adversarial roles themselves, as in bug bounties or games, what are the limits of acceptable behaviour?

Although specific guidelines exist for penetration testing and bug bounties, the general question of how informed consent can be reconciled with adversarial roles in the experiment still needs to be answered for the case where experiments are real deployments.

### Learning

Learning is an important goal of security-by-experiment.^4^ In essence, one tries to induce learning from incidents in pilots (Drupsteen and Guldenmund 2014) before real large-scale incidents occur.

In this context, there is one important aspect for discussion. When we enable learning for cyber security by means of social experiments, it may not only be the defenders who learn, *but also the adversaries*. These can be external adversaries, or participants in the experiments who have been assigned adversarial roles and then misuse what they learnt later. This is the same issue that we considered when letting students play adversarial roles (Dimkov et al. 2011). In other words, aren’t we providing adversaries with malicious ideas when experimenting with security-sensitive technologies, designing adversarial aspects into such experiments, and trying to learn from those? One could argue that hiding such information from adversaries would not be possible in the long run anyway, but still the experiment may require an additional condition, stating that learning by potential adversaries should be minimised in the design of the experiment.

In particular, (i) a set of criteria should be established on who is eligible for assigned adversarial roles, (ii) the adversaries should have anonymous means of raising questions and concerns during the experiment, and (iii) the adversaries should participate in an evaluation session afterwards. Finally, as it should not be assumed that the assigned roles are the only adversaries active in the experiment, a discussion on who else might have acted as an adversary and/or obtained information that would enable this in the future (NGOs, but also criminal organisations) should be part of the evaluation.

### Governance

In addition, there is the question whether deployment experiments in the cyber world need more high-level guidance. We have seen in the Dutch examples that each deployment seems to be rather ad-hoc, without clear guidance on evaluating security while scaling up. Several people have suggested to us that institutional arrangements, such as the FDA (US Food and Drugs Administration) in case of drugs, might assist in developing knowledge and guidance on deployment processes and associated experimental practices. Maybe national cyber security centres could play a similar role for cyberspace, and there may be similar options for other security-sensitive technologies. Although this is undoubtedly controversial, and it is not our core field of study, we think that the question would be worth discussing from a policy point of view.

In this light, it is interesting to observe that new regulation on cyber security is already on its way, for example in the form of the EU Directive on Network and Information Security. Responsible deployment, in the form of “security-by-experiment” could have a place in the implementation of the Directive by the member states, next to the commonly advocated “security-by-design”.

## Conclusions and Discussion

In our earlier paper (Pieters et al. 2014a), we highlighted for the cyber security community how NTaSE could provide a useful perspective on deployment of security sensitive technologies in the real world. The technological infrastructure and socio-political context is simply too complex for security assessments to be able to predict all potential side-effects and challenges the technology will face. So we argued, in line with NTaSE, that deployment of security-sensitive technologies should be regarded as an experiment, and discussed how conditions for responsible experimentation from the paradigm may be used to address issues that arise in the cyber security setting. For example, the condition of feedback points to the observation that feedback may also be available to adversaries. Similarly, the condition of conscious scaling up points to the observation that larger-scale systems become more attractive for adversaries.

In the current contribution, conversely, we have investigated what it would mean for the NTaSE paradigm to take security aspects into account next to safety. We did so based on an overview of experimental and responsible deployment approaches in the domain of cyber security, drawing lessons from the concept of adversarial roles in such approaches (Table 1). These approaches come at different costs, and may not be feasible for all types of systems. A careful selection of applicable methods is therefore needed when a deployment plan is made.

One open issue is that with respect to adversarial roles, different adversaries may be interested in different outcomes. For example, individual travellers may try to clone their own OV-chipkaart, criminal organisations may be interested in selling large numbers of cloned cards, marketing firms may be interested in profiling customers based on their electricity consumption, and hostile foreign governments may be interested in influencing the outcome of an election. The question is to what extent adversarial roles in security-by-experiment need to represent those different adversaries, and if so, how this could be achieved in practice. At the very least, we can assume that the *goals* of the adversarial roles will influence the outcome of the experiment, and we should be aware of this in the evaluation.

With respect to learning, a balance needs to be sought between the adversarial roles that are foreseen in providing feedback on security issues, and the increase in security risk induced by the publication of information that enables such feedback. In cyber security, there is a general tendency towards openness of designs (if not software), based on Kerckhoffs’ principle. Following that principle, “security by obscurity” has been criticised as bad practice, although there have been some efforts to rehabilitate obscurity as a sensible security control (Pavlovic et al. 2011; Stuttard 2005).

As for the importance of adversarial aspects, these are of course broader than NTaSE only. Security-by-design and privacy-by-design require adversarial perspectives as well, as exemplified by security testing as opposed to regular testing. Even moral principles related to risk may have different interpretations in an adversarial context, for example the precautionary principle (Pieters and Van Cleeff 2009), primarily because at least part of the responsibility for the effects seems to lie with the adversary rather than the designer. Thus, the adversarial question extends to the whole risk management chain (Rios Insua et al. 2009), from principles to the evaluation of social experiments.

In a sense, we have also taken a somewhat adversarial role in this paper with respect to the deployment of the NTaSE paradigm, pointing to a vulnerability that may give adversaries an advantage. We hope that our adversarial role was scripted carefully enough to prevent harm, and to enable some learning indeed.

## Footnotes

1 The electronic voting and smart metering examples are the same as in the earlier conference paper (Pieters et al. 2014a).

2 http://www.sos.cs.ru.nl/applications/rfid/pressrelease-courtdecision.en.html.

3 Thanks to Demetris Antoniou for this suggestion.

4 This subsection is largely the same as in the preceding conference paper (Pieters et al. 2014a).
